# Investigation of wavy microchannel ability on electronic devices cooling with the case study of choosing the most efficient microchannel pattern

**DOI:** 10.1038/s41598-022-09859-6

**Published:** 2022-04-07

**Authors:** Nima Ghorbani, Mohammad Zabetian Targhi, Mohammad Mahdi Heyhat, Yousef Alihosseini

**Affiliations:** Faculty of Mechanical Engineering, Tarbiat Modares University, Tehran, Iran

**Keywords:** Energy science and technology, Engineering

## Abstract

A numerical study was conducted to investigate the ability of wavy microchannels to damp the temperature fluctuations generates in electronic devices. Five wavy patterns are considered with the amplitude and wavelength in the ranges of 62.5 to 250 μm and 1250 to 5000 μm, respectively to study the effect of governing phenomena of flow within wavy patterns on thermal–hydraulic performance. The flow regime is laminar and the Reynolds number is in the range of 300 to 900, and a relatively high heat flux of 80 W/cm^2^ is applied to the microchannels substrate. Also, variable flux condition is studied for heat fluxes of 80, 120, 160, 200, and 240 W/cm^2^ and for the most efficient wavy and straight microchannels. Results showed that the geometries with larger amplitude to wavelength ratio have a lower radius of curvature and larger Dean number, and as a result of transverse flow (secondary flow) amplification, they have enhanced heat transfer. Also, by comparing the ratio of the transverse velocity components to the axial component, it was found that by decreasing the radius of curvature and increasing the Dean number, transverse velocity increases, which intensifies the heat transfer between the wall and the fluid. The appraisement of the performance evaluation criterion (PEC) illustrates that the wavy case with an amplitude of 250 μm and wavelength of 2500 μm is the best geometry from the thermal–hydraulic point of view in the studied range. Finally, with variable flux condition, the wavy microchannel has responded well to the temperature increase and has created a much more uniform surface temperature compared to straight pattern. The proposed wavy pattern ensures that there are no hotspots which could damage the electronic chip. Presented wavy patterns can be used in heat sinks heat transfer enhancement to allow the chip to run in higher heat fluxes.

## Introduction

With the ever-increasing progress of the electronics industry towards higher and faster processing power systems, electronic cooling has always been one of the essential issues in this industry. In recent years, the need for more efficient cooling systems with a higher heat transfer rate has been considered. Various approaches have been proposed for electronic cooling, among which microchannel heat sink (MCHS) is one of the most efficient ones. Tuckerman and Pease^1^ proposed the idea of using the microchannel heat sink in 1981. Since then, numerous studies have been carried out on how to enhance the heat transfer in these systems. The use of particulate fluids (nanofluid, e.g.)^2,3^, the use of different manifolds shape^4,5^, changing shape of channel cross-section^6,7^, and changing channel geometry^8,9^ are among the methods that have been studied in recent years to improve the thermal performance of these systems. Recently, critical researches were performed about the use of nanofluids, and the effect of geometric design on the microchannel heat sinks thermal–hydraulic performance^10–13^.

In the laminar flow range, the convective heat transfer significantly enhanced by enhancing fluid mixing. The flow of fluid through curved passages due to the presence of centrifugal force increases the fluid mixing and decreases the thickness of the thermal and hydrodynamic boundary layers and thereby enhances the heat transfer. Among the curved passages, the trigonometric wavy geometries due to their better thermal–hydraulic performance than the other curved passages, have engrossed much consideration in last years.

A critical study was conducted by Sui et al.^14^ regarding fluid flow in wavy microchannels. The results demonstrated the higher performance of the wavy microchannels compared with the straight case. They reported that Dean vortices generation led to chaotic advection and fluid mixing which improves the heat transfer in these geometries. They also concluded that increasing the amplitude to wavelength ratio in the flow path enhances the performance of these systems to remove the higher heat fluxes and prevent the generation of hotspots. In another study by the same research group^15^, they evaluated the performance of wavy microchannels experimentally and numerically. The results showed that these microchannels significantly enhance heat transfer compared to straight microchannels due to the formation of Dean vortices. The pressure drop penalty in these geometries is much lower than the augmentation of heat transfer, for instance, for the wavy microchannel with the amplitude of 259 μm, the friction factor increased by 76%, while the heat transfer coefficient increased by 211%. After Sui et al.^14,15^, heat transfer enhancement in wavy microchannel heat sink was also numerically investigated by Mohammed et al.^16^. They considered five wavy geometries with different amplitudes and concluded that increasing the amplitude does not always lead to an increase in heat transfer and there is an optimal limit for it. In the other work^17^, they analyzed the influence of channel shape for three shapes, include zigzag, curvy, and step, and compared the heat transfer coefficient with that of straight and wavy microchannels. The results revealed that the zigzag MCHS had the best thermal performance, followed by wavy, curvy, and step channels, respectively. The zigzag pattern also had the highest pressure drop and friction factor. They concluded that due to the generation of swirl, eddy, and recirculation flows in the zigzag geometry, it has the highest heat transfer and pressure drop compared with the other shapes mentioned above. Similar to the results of the previous studies, Gong et al.^18^ detected that the wavy microchannels have a higher thermal performance compared with the straight geometry. It enhances with increasing the Reynolds number and the wavy amplitude and decreasing the wavelength.

Since then, researchers have conducted most of their studies to improve and optimize the heat transfer in this geometry, as well as comparing it with other periodic geometries. Rostami et al.^19^ conducted a numerical investigation to found an optimum geometry for wavy walls microchannels. They addressed that due to the existence of secondary flows and recirculation zones in wavy geometries, the heat transfer of these microchannels enhanced by increasing the Reynolds number and wavy amplitude. Also, they reported that there is an optimum geometry for wavy wall microchannels that the heat transfer would be maximum. They highlighted that the dependence of vortices shape and intensity to the Reynolds number and geometrical parameters is the reason for existing an optimum mode for these geometries. The efficacy of corrugation shape on a corrugated minichannel heat sink performance was analyzed experimentally and numerically by Aliabadi et al.^20^. They considered sinusoidal, triangular and trapezoidal patterns, and compared with each other and with the conventional straight geometry. The results represented the maximum values of Nusselt number and pressure drop for the trapezoidal pattern. In order to evaluation of the thermal–hydraulic performance of the mentioned geometries, they calculated the heat transfer rate to pumping power ratio and addressed that the sinusoidal channel has the best overall performance. Lin et al.^21^ in a numerical study declared that by increasing the absolute difference between the amplitude or wavelength of two contiguous wavy units, the enhancement of heat transfer could be more significant. Parlak et al.^22^ compared heat transfer characteristics of wavy microchannel with zigzag and straight geometries numerically. They inferred that the Nusselt number of wavy microchannel is 10% and 40% higher than those of zigzag and straight microchannel, respectively. Huang et al.^23^ conducted an experimental study and explored that the phase difference between two parallel walls of the wavy microchannels could improve the thermal performance of these geometries. Also, Nakhchi^24^ presents an optimization of geometrical parameters inside sinusoidal wavy channels and revealed that the heat transfer to pressure drop ratio is maximum for wave’s amplitude ratio of 0.54, phase shift of 0, and the number of waves of 11. Zhu et al.^25^ investigated performance comparison of wavy microchannel heat sinks with left–right and up-down designs and concluded that the up-down design represents a better heat transfer performance at small wavelengths and at large wavelengths, both designs perform the same. Valaparla et al.^26^ studied the effect of sidewall rib on Heat Transfer and Fluid Flow Characteristics of wavy microchannel and revealed that thermal resistance decreased by 33.6% for the wavy microchannel with rib. Although this improvement of heat transfer, accompanied with pressure drop penalty. Optimization of wavy microchannel with grooves is studied numerically by Park et al.^27^. They considered three parameters as design variable including the distance between staggered grooves, groove width, and groove depth and inferred that thermal resistance and friction factor of optimal design improved by 1.55% and 3%, respectively. Wang et al.^28^ conducted a numerical study of heat transfer enhancement of symmetric and parallel wavy microchannel heat sinks with secondary branch and deduced that for the wavy geometries without secondary branch, the overall performance of the symmetric geometry is slightly lower than that of the parallel geometry. Also, they concluded that due to secondary branches, the suction effect at channel throats in symmetric wavy configuration weaken the Dean vortices and result in heat transfer decrement.

Recently, interrupted, crosscut flow, and non-uniform wavy patterns have been further perused because of improved thermal–hydraulic performance, and some of the prominent studies are found in the following references^29–33^. Also, many researches are done on the wavy patterns regarding utilization of the wavy geometries and nanofluids, simultaneously^34–36^.

By reviewing some of the important studies conducted in this field, in the present study, to cover a wide range of geometries, five wavy geometries have been considered with different amplitudes and wavelengths. The fluid flow and heat transfer in these geometries have been simulated numerically in the laminar flow range and the Reynolds number of 300 to 900 and the heat flux of 80 W/cm^2^. While the thermal–hydraulic performance of the mentioned geometries has been evaluated by the performance evaluation criterion to introduce the most efficient case, the role of Dean number and radius of curvature on the generation of the secondary flows and increasing the transverse velocities and the relation between these parameters in a wide range of amplitude and wavelength of wavy microchannels are investigated which are considered as the first novelty of this work. Meanwhile, a critical study of variable flux condition which is the second innovation of the present work are conducted at the Reynolds number of 900 and for the most efficient wavy microchannel in heat fluxes of 80, 120, 160, 200, and 240 W/cm^2^ to clarify the ability of the wavy microchannels to prevent the generation of hotspots and chip damage.

## Geometry of microchannels

**Figure 1:**
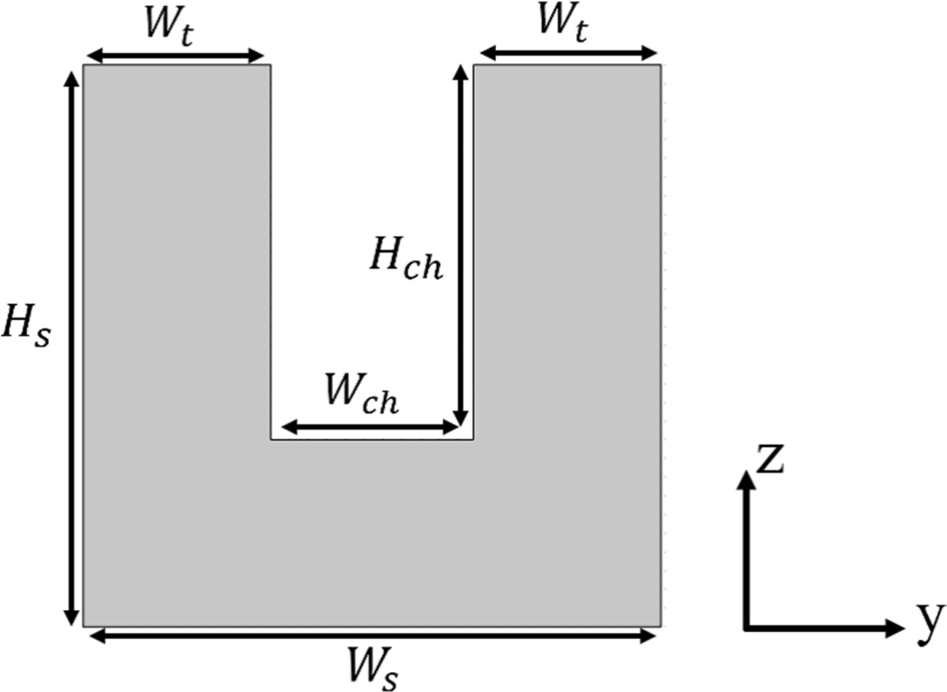
The cross-section of studied microchannels.

**Figure 2:**
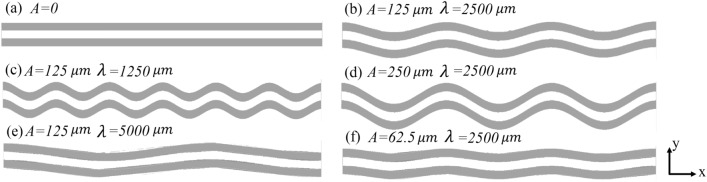
The top view of a part of (**a**) Straight microchannel, (**b**) Wavy case 1, (**c**) Wavy case 2, (**d**) Wavy case 3, (**e**) Wavy case 4 and (**f**) Wavy case 5.

In this work, all of the studied microchannels have a width of 216 μm, a height of 400 μm, and a length of 25 mm. Also, copper substrate thickness is considered constant and equal to 200 μm. Figure 1 shows the cross-section of the investigated microchannels and the dimensions of the studied microchannels are summarized in Table 1. Five wavy-type geometries were generated based on the trigonometric profile of $y = A{\text{Cos}} ({{2\pi x} \mathord{\left/ {\vphantom {{2\pi x} \lambda }} \right. \kern-\nulldelimiterspace} \lambda })$ that A is the wavy amplitude, and λ is the wavelength. The geometrical specifications of these microchannels are given in Table 2, and the top view of a part of the channels is depicted in Fig. 2. According to a review of some of the prominent studies in this regard, the wavy geometry number 1 with an amplitude of 125 μm and a wavelength of 2500 μm is considered as a reference wavy geometry^15,16^, and four other geometries created with a change in the amplitude (cases 3, and 5) or wavelength (cases 2, and 4) relative to case 1. these geometries have been created in such a way that in two cases, the amplitude to wavelength ratio is doubled (cases 2, and 3) and in two cases, the amplitude to wavelength ratio is halved (cases 4, and 5).

**Table 1:** Dimensions of the computational domain.

$H_{ch}$ (μm)	$W_{ch}$ (μm)	$H_{s}$ (μm)	$W_{s}$ (μm)	$W_{t}$ (μm)	$L_{ch}$ (mm)
400	216	600	616	200	25

**Table 2:** Geometrical specifications of the wavy microchannels.

Wavy geometries	*A* (μm)	*λ* (μm)	$\frac{A}{\lambda }$	$r_{c}$ (μm)
Case 1 (Ref.)	125	2500	0.05	1266.5
Case 2	125	1250	0.1	316.6
Case 3	250	2500	0.1	633.2
Case 4	125	5000	0.025	5066.1
Case 5	62.5	2500	0.025	2533.1

In the present study, the substrate material and the working fluid respectively considered copper and water which the properties of these materials are listed in Table 3.

**Table 3:** Thermophysical properties of the working fluid and substrate material^37^.

Material	$\rho \; ({\text{kg}}/{\text{m}}^{3} )$	$c_{p} \; ({\text{J}}/{\text{kg}}\,{\text{K}})$	*k *(W/mK)	*μ *(kg/ms)
Water	998.2	4184	0.6	0.0010016
Copper	8933	385	401	***

## Numerical modeling

### Governing equations

Three-dimensional simulation of flow in microchannel is carried out by conjugate heat transfer method to consider both conduction in the solid domain and convection in the fluid domain. The flow in this simulation is considered incompressible, and is laminar according to the range of the Reynolds number. It is noteworthy that Kandlikar et al.^38^ declare that laminar to turbulent transition in microchannels occurs around Reynolds number of 2300. Moreover, as mentioned in “Introduction”, a prominent experimental study by Sui et al.^15^ investigated the wavy microchannel heat sink in the range of Re = 300 to 800 and explain that the flow regime is laminar in both of straight and wavy microchannels. Also, Khoshvaght-Aliabadi et al.^39^ studied the enhancement of laminar forced convection cooling in wavy heat sink in the range of Re = 100 to 900 and declare that the flow regime is laminar. There are many references that proved there is no significant deference between the large scale and micro scale transition Reynolds number. So, the flow regime in the present study (Re = 100 to 900) is also considered laminar. The other assumptions are taken into account as following: (1) constant fluid and solid properties (due to low temperature changes), (2) negligible gravitational force, (3) neglected viscous dissipation, (4) neglected heat loss between the microchannel and the ambient, and (5) neglected radiative and natural convective heat transfer. Based on the assumption expressed, the governing equations are defined as follows^21^:

Continuity equation:

1 $$\rho_{f} \nabla .\left( {\vec{U}} \right) = 0.$$

Momentum equation:

2 $$\rho_{f} \frac{{\partial \vec{U}}}{\partial t} + \rho_{f} \left( {\vec{U}.\nabla } \right)\vec{U} = - \nabla p + \mu \nabla^{2} \vec{U}.$$

Energy equation for the fluid:

3 $$\rho_{f} C_{{p_{f} }} \frac{\partial T}{{\partial t}} + \rho_{f} C_{{p_{f} }} \left( {\vec{U}.\nabla T} \right) = k_{f} \nabla^{2} T.$$

Energy equation for the solid:

4 $$\rho_{s} C_{{P_{s} }} \frac{\partial T}{{\partial t}} = k_{s} \mathop \nabla \nolimits^{2} T.$$

In the presented equations, *p* is the pressure, $\vec{U}$ is the velocity vector, $\rho_{f}$, *μ*, $C_{{p_{f} }}$, and $k_{f}$ are respectively the fluid density, dynamic viscosity, specific heat, and thermal conductivity, and $\rho_{s}$, $C_{{p_{s} }}$, and $k_{s}$ are the solid density, specific heat, and thermal conductivity.

### Boundary conditions

**Figure 3:**
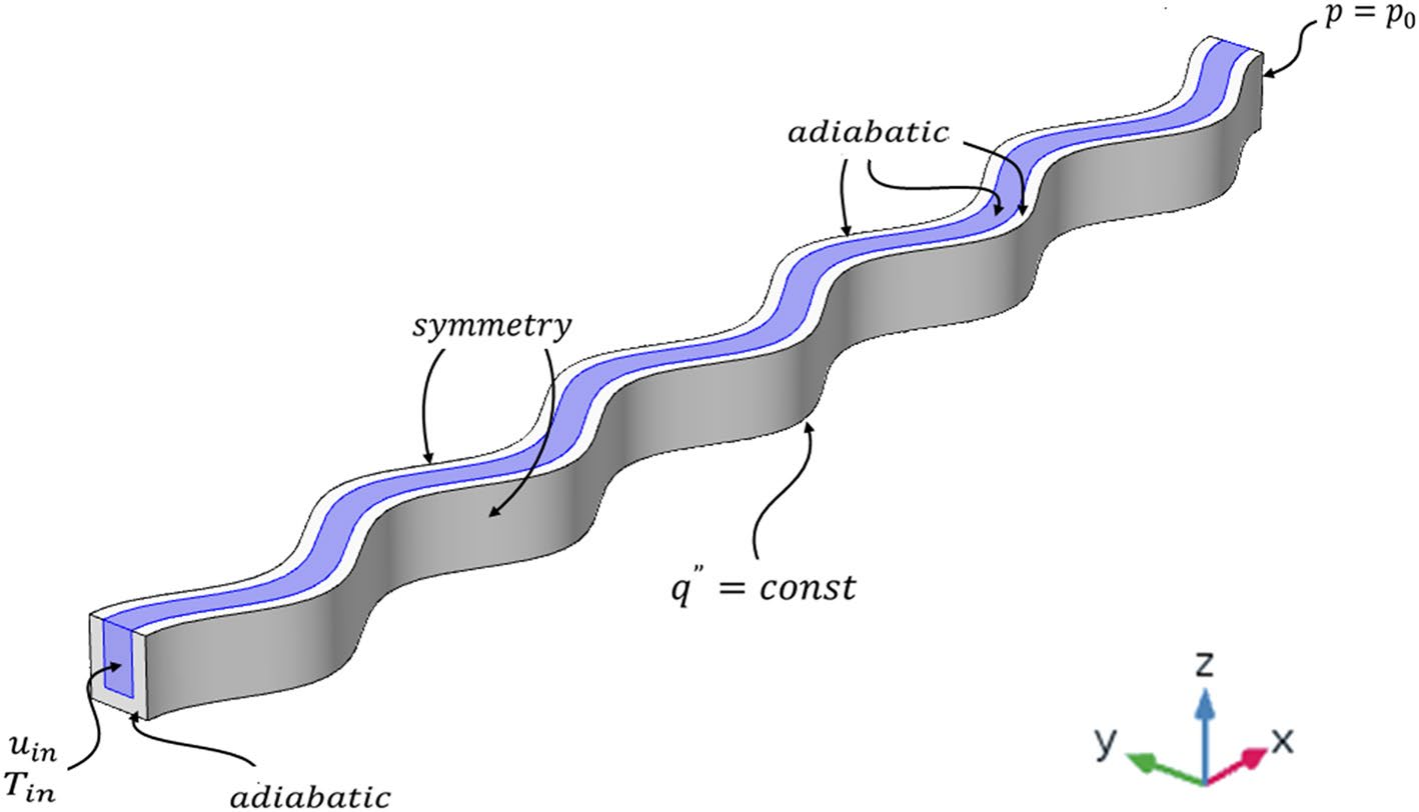
Schematic of wavy channel and the desired boundary conditions.

The boundary conditions are expressed as: the fluid velocity and temperature are assumed to be constant at the inlet, and the pressure at the outlet is specified as atmospheric pressure. Meanwhile, a constant heat flux is exerted to the bottom surface of microchannel, and the boundary condition at the side walls is considered as symmetry. The adiabatic boundary condition is utilized at the other outer surfaces of the heat sink substrate that is compatible to the real situation of proper isolation. At interface of the solid and fluid, the solid and fluid temperatures assumed equal and also, the heat flux is considered to be continuous. Based on the no-slip condition, the velocity on the microchannel walls is zero. So, the boundary conditions are as follows and presented in Fig. 3 and Table 4^21^.

**Table 4:** Boundary conditions.

At the inlet	At the outlet	At the substrate bottom surface	At the fluid–solid interface	Other outer surfaces
$u = u_{in}$ $v = w = 0$	$p = p_{0}$	***	$u = v = w = 0$	***
$T = T_{f,in}$	***	***	$T_{s} = T_{f}$	***
***	***	$q^{{{\prime\prime}}} = - k_{s} \frac{{\partial T_{s} }}{\partial n} = const$	$k_{s} \frac{{\partial T_{s} }}{\partial n} = k_{f} \frac{{\partial T_{f} }}{\partial n}$	$q^{{{\prime\prime}}} = - k_{s} \frac{{\partial T_{s} }}{\partial n} = 0$

where *u*, *v*, and *w* represent the components of the velocity vector in the *x*, *y*, and *z* directions, $u_{in}$ is the fluid inlet velocity, $T_{f,in}$ is the fluid inlet temperature, $p_{0}$ denotes the atmospheric pressure, *q”* indicates the heat flux exerted to the bottom surface of the substrate, $T_{s}$, and $T_{f}$ are the solid and fluid temperatures, and *n* is the perpendicular direction to the surface.

### Performance evaluation parameters

In order to assess the performance of the heat sink, performance evaluation parameters such as the average Nusselt number, friction factor, performance evaluation criterion, and Dean number are investigated, which their formulations are presented as follows. The first performance indicator is the average Nusselt number that is given by^40^:

5 $$Nu_{avg} = \frac{1}{{L_{ch} }}\mathop \smallint \limits_{{L_{ch} }} Nu_{x} dx,$$

where $L_{ch}$ is the length of the channel and $Nu_{x}$ is the local Nusselt number that is defined as:

6 $$Nu_{x} = \frac{{h_{x} D_{h} }}{{k_{f} }},$$

where $k_{f}$ is the fluid thermal conductivity, and $D_{h}$ is the channel hydraulic diameter that is defined by:

7 $$D_{h} = \frac{{4A_{c} }}{{P_{c} }},$$

where, $A_{c}$ is the cross-section area of microchannel and $P_{c}$ is the perimeter of microchannel cross-section:

8 $$A_{c} = W_{ch} \times H_{ch} ,$$

9 $$P_{c} = 2(W_{ch} + H_{ch} ).$$

In Eq. (6)$h_{x}$ is the local heat transfer coefficient that is calculated from^40^:

10 $$h_{x} = \frac{{q^{\prime\prime} A_{b} }}{{\left( {T_{W,x} - T_{f,x} } \right) A_{i} }},$$

where $q^{\prime\prime}$ is the heat flux, $A_{b}$ is the base area of the channel that the heat flux applied to it, $A_{i}$ is the fluid–solid interface area that the coolant removes the heat from it, while $T_{W,x}$ and $T_{f,x}$ are the local wall temperature and the local fluid bulk temperature. $A_{b}$, $A_{i}$, $T_{W,x}$, and $T_{f,x}$ can be expressed by Eqs. (11) to (14), respectively^40^ (Geometrical parameters can be seen in Fig. 1).

11 $$A_{b} = (W_{ch} + 2W_{t} )L_{ch} = W_{s} L_{ch} ,$$

12 $$A_{i} = (W_{ch} + 2H_{ch} )L_{ch} ,$$

13 $$T_{W,x} = \frac{1}{W}\mathop \smallint \limits_{W} T_{W} dW,$$

14 $$T_{f,x} = \frac{{\mathop \smallint \nolimits_{{A_{c} }} \rho_{f} uC_{{p_{f} }} T_{f} dA_{c} }}{{\mathop \smallint \nolimits_{{A_{c} }} \rho_{f} uC_{{p_{f} }} dA_{c} }},$$

where $W_{ch}$ is width of channel, $W_{t}$ is thickness of substrate, $W_{s}$ is width of substrate, $L_{ch}$ is length of channel, $H_{ch}$ is height of channel, $\rho_{f}$ is density of the fluid, $C_{{p_{f} }}$ is heat capacity of the fluid, *u* is the fluid velocity, $T_{f}$ is the fluid temperature, and $A_{c}$ is the cross-section of the channel.

The friction factor is one of the main hydraulic performance indicators that is evaluated by^40^:

15 $$f = \frac{{2\Delta PD_{h} }}{{\rho_{f} L_{ch} u_{avg}^{2} }},$$

where $\Delta P$ represents the pressure drop of the channel and $u_{avg}$ is the average longitudinal velocity that is given by:

16 $$u_{avg} = \frac{{\mathop \smallint \nolimits_{{A_{c} }} u dA_{c} }}{{A_{c} }}.$$

In the direction of evaluate the increase of the Nusselt number and friction factor of the wavy patterns respect to the straight pattern, two parameter of the Nusselt number enhancement and friction factor enhancement are expressed as follows^14,41^:

17 $$E_{Nu} = \frac{{Nu_{Wavy} }}{{Nu_{Straight} }},$$

18 $$E_{f} = \frac{{f_{Wavy} }}{{f_{Straight} }}.$$

One of the dimensionless numbers defined in curved paths to evaluate the intensity of the transverse flows is the Dean number. In the present study, the relationship between the geometrical characteristics of the channels and velocity vectors in the cross-sections of the channels with the help of Dean number is investigated that is defined by^42–44^:

19 $$De = {\text{Re}} \sqrt {\frac{{D_{h} }}{{2R_{C} }}} ,$$

where Re is the Reynolds number, and $R_{C}$ is the radius of curvature that are given by Eqs. (20) and (21):

20 $${\text{Re}} = \frac{{\rho u_{avg} D_{h} }}{\mu },$$

21 $${\text{R}}_{C} = \frac{{(1 + (dy/dx)^{2} )^{3/2} }}{{|d^{2} y/dx^{2} |}}.$$

As mentioned in “Geometry of microchannels”, wavy geometries were generated based on the trigonometric profile of $y = A{\text{Cos}} ({{2\pi x} \mathord{\left/ {\vphantom {{2\pi x} \lambda }} \right. \kern-\nulldelimiterspace} \lambda })$ that A is the wavy amplitude, and λ is the wavelength. So in order to calculate $R_{C}$ we have:

22 $$dy/dx = \left( { - \frac{2\pi }{\lambda }A} \right){\text{Sin}} \left( {\frac{2\pi x}{\lambda }} \right),$$

23 $$d^{2} y/dx^{2} = - \left( {\frac{2\pi }{\lambda }} \right)^{2} A{\text{Cos}} \left( {\frac{2\pi x}{\lambda }} \right).$$

In the present study $R_{C}$ is calculated at extremum point of $y = A{\text{Cos}} ({{2\pi x} \mathord{\left/ {\vphantom {{2\pi x} \lambda }} \right. \kern-\nulldelimiterspace} \lambda })$, which consequently result in minimum $R_{C}$ and maximum Dean number. Also, at the extremum point, x is a multiple of $\lambda /2$ and therefore ${\text{Sin}} ({{2\pi x} \mathord{\left/ {\vphantom {{2\pi x} \lambda }} \right. \kern-\nulldelimiterspace} \lambda })$ is zero and ${\text{Cos}} ({{2\pi x} \mathord{\left/ {\vphantom {{2\pi x} \lambda }} \right. \kern-\nulldelimiterspace} \lambda })$ is equal to 1.

In pursuance of investigate both of the thermal and hydraulic performance at the same time or in other words the overall performance of the heat sink, the performance evaluation criteria are defined by Eqs. (24) and (25) ^45^:

24 $$PEC_{Wav/Str} = \frac{{Nu_{Wavy} /Nu_{Straight} }}{{(f_{Wavy} /f_{Straight} )^{1/3} }},$$

25 $$PEC_{Wav/Wav,REF} = \frac{{Nu_{Wavy} /Nu_{Wavy,REF} }}{{(f_{Wavy} /f_{Wavy,REF} )^{1/3} }},$$

where $PEC_{Wav/Str}$, and $PEC_{Wav/Wav,REF}$ are the performance evaluation criteria for the wavy patterns respect to the straight pattern, and reference wavy pattern, respectively. Equation (24) compares the wavy patterns with the straight pattern to introduce the best geometry from thermal–hydraulic point of view. Equation (25) compares the wavy patterns with the reference wavy pattern to answer to the question that does the wavy pattern always increased the thermal–hydraulic performance.

### Numerical validation

The microchannel geometry is generated in GAMBIT software and meshed in structured form. The governing equations are solved using finite volume based Ansys Fluent software. The SIMPLE scheme is applied for pressure–velocity coupling. The second-order-upwind-scheme is used to solve the momentum and energy equations, and the standard scheme is employed for pressure discretization. In order to empower the precision of the results, solving convergence is considered when the residuals of the continuity and momentum equations are less than 10^–8^ and the residual of the energy equation is less than 10^–10^.

**Figure 4:**
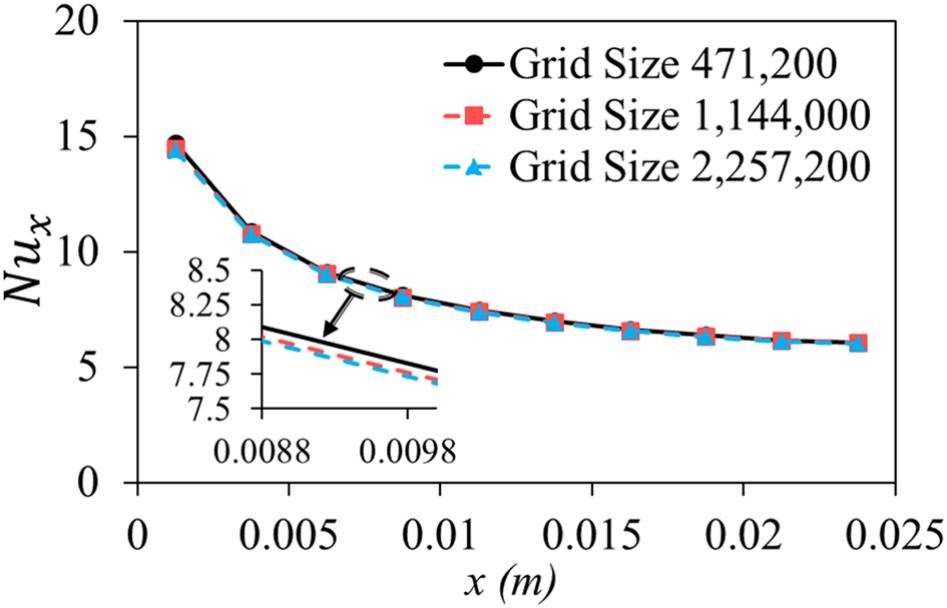
The local Nusselt number for grid independency examination.

**Figure 5:**
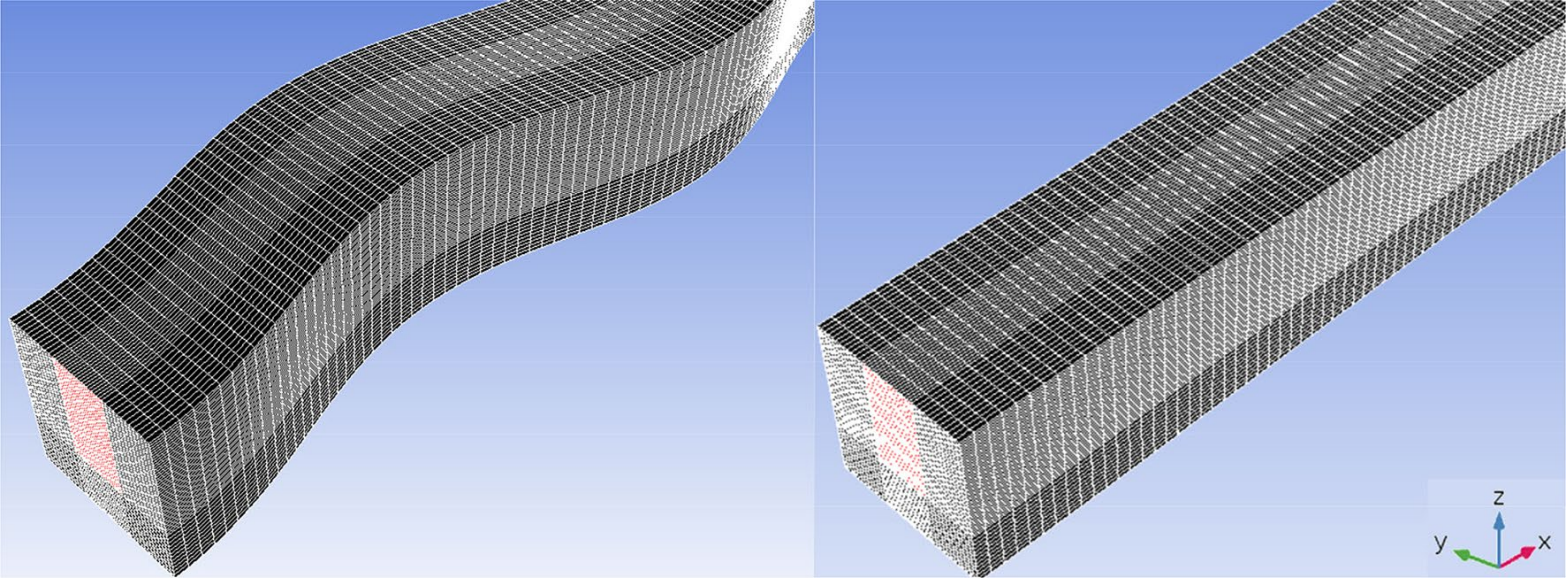
A view of generated mesh for both straight and wavy geometries.

To ensure the grid independency of the solution, three computational grids are considered with the mesh density of $(y \left(channel \; width\right)\times z \left(channel \; height\right) \times x \left(channel \; length\right))$ 31 × 38 × 400, 44 × 52 × 500, and 57 × 66 × 600. The Nusselt number as the main indicator of the heat exchanger performance is calculated for each mentioned grid and presented in Fig. 4. The average difference between the average Nusselt number of the first and third grid results is 1.31% that with a further increase in the size of the grid and for the second and third grids this difference has declined to 0.4%. According to the precision of the results as well as saving computational time and costs, the second grid (with size of 1,144,000 cells) is employed in this study. Figure 5 illustrates the utilized mesh for both straight and wavy geometries.

For the validation of the present work, the results of the experimental study by Sui et al.^15^ are used to compare with present simulation. In the experiment that carried out by the study, a microchannel heat sink with 62 microchannels was investigated. The width, height, and length of each microchannel were 206 μm, 408 μm, and 25 mm, respectively, and the wall thickness between two parallel microchannels was 194 μm.

**Figure 6:**
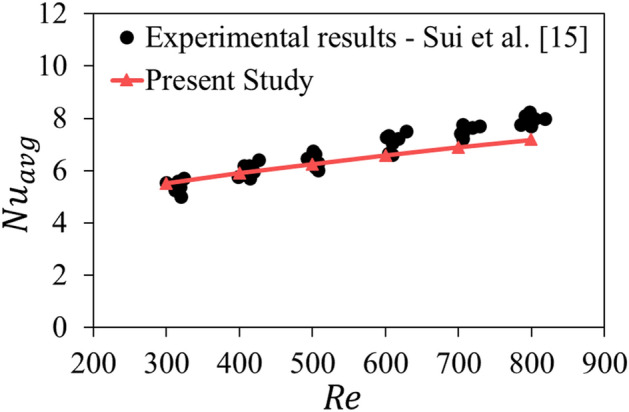
Validation of the numerical model (average Nusselt number versus Reynolds number).

Their study was performed for the Reynolds number in the range of 300 to 800 and heat flux of 50 W/cm^2^. As can be seen in Fig. 6, the results of the simulation have an acceptable agreement with the reference results. The maximum difference of the simulation results with the average of the experimental data for the average Nusselt number is 9.6% and the minimum is 1.9% for the Reynolds number of 900 and 500, respectively. Based on this comparison, the validity of the results of the present study is ensured.

## Results and discussion

In the present study, the substrate material and the working fluid respectively considered copper and water which the properties of these materials are listed in Table 3. In the “Efficacy of the geometrical parameters on the performance indicators”, “The relation between the intensity of the transverse flow, heat transfer and the Dean number”, and “Performance evaluation criterion”, the simulation was conducted at steady state condition and the considerable heat flux of 80 W/cm^2^ that selected based on the literature review, the Reynolds number of 300, 500, 700, and 900 with the inlet temperature of 293.15 K. In the “Comparison of the optimal wavy channel with the straight channel in variable flux conditions”, with transient simulation, the variable heat flux condition is investigated for heat fluxes of 80, 120, 160, 200, and 240 W/cm^2^ and the Reynolds number of 900, which leads highest heat transfer rate in the present study flow rate range.

### Efficacy of the geometrical parameters on the performance indicators

**Figure 7:**
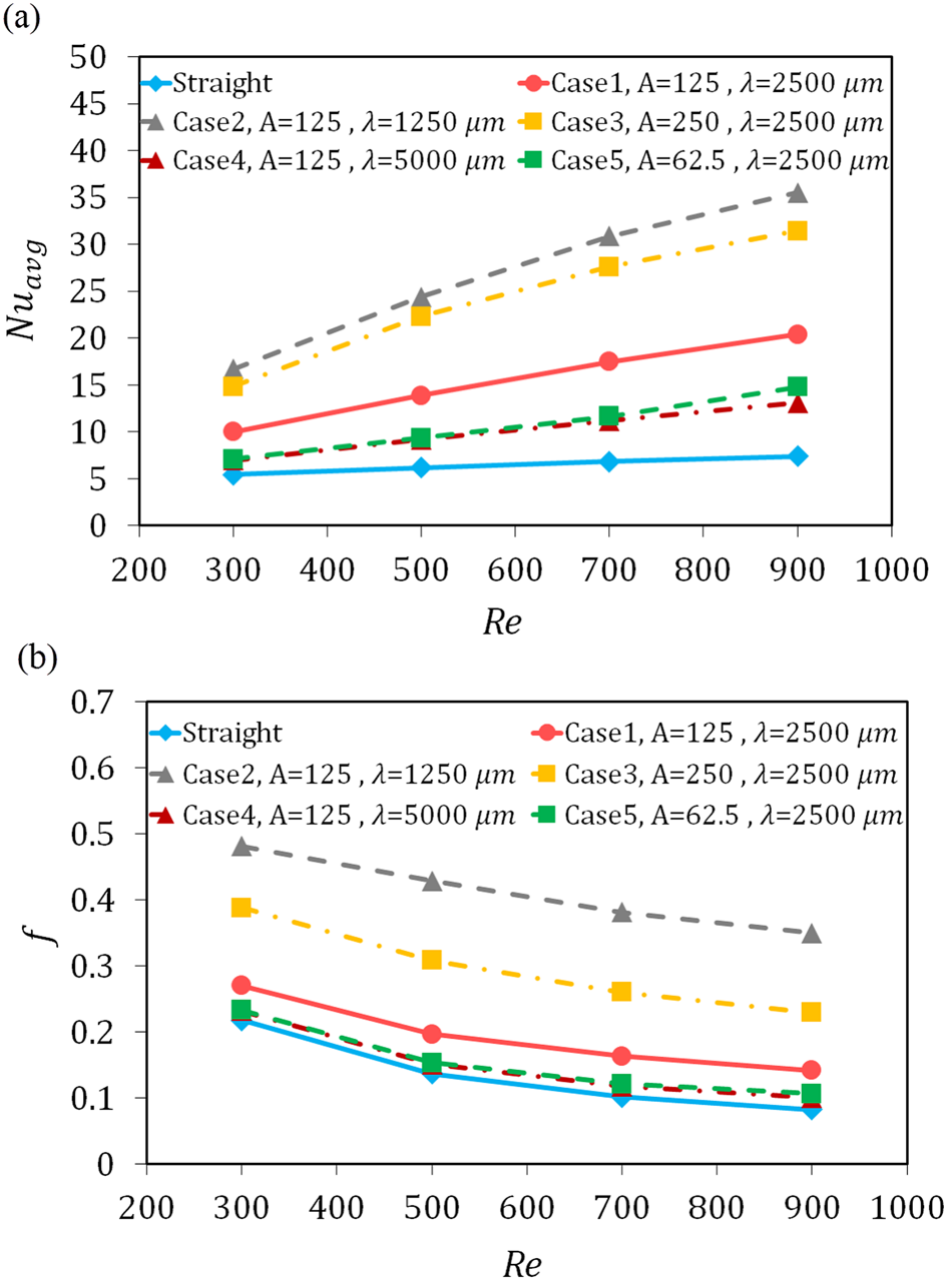
Comparison for (**a**) the average Nusselt number, and (**b**) friction factor versus the Reynolds number.

In this section, the effects of changing the amplitude and the wavelength of the wavy microchannel are assessed with the performance indicators and enhancement of these parameters, *Nu*_*avg*_ and *f*. average Nusselt number and friction factor versus the ReynoldsFigure 7 shows the number for all five wavy cases in comparison with those of straight microchannel. As can be seen, for all wavy geometries the values ​​of these two parameters are higher than their values ​​for straight geometry, and case 2 had the highest values, followed by cases 3, 1, 5 and 4, respectively. It is clear that increasing the amplitude and decreasing the wavelength, or in other words, increasing the amplitude to wavelength ratio, has increased the heat transfer in these systems, which is accompanied by an increase in pressure drop. Also, wavy geometries with the same amplitude to wavelength ratio have thermal and hydraulic performance close to each other. However, wavy geometries created by changing the wavelength (case 2 and case 4) relative to the reference case (case 1) had a more significant effect on increasing or decreasing heat transfer. Another noteworthy point is that according to the Table 2 and Fig. 7, wavy microchannels with a smaller radius of curvature show better thermal performance, which is accompanied by a higher pressure drop. This issue is discussed further in “The relation between the intensity of the transverse flow, heat transfer and the Dean number”.

**Figure 8:**
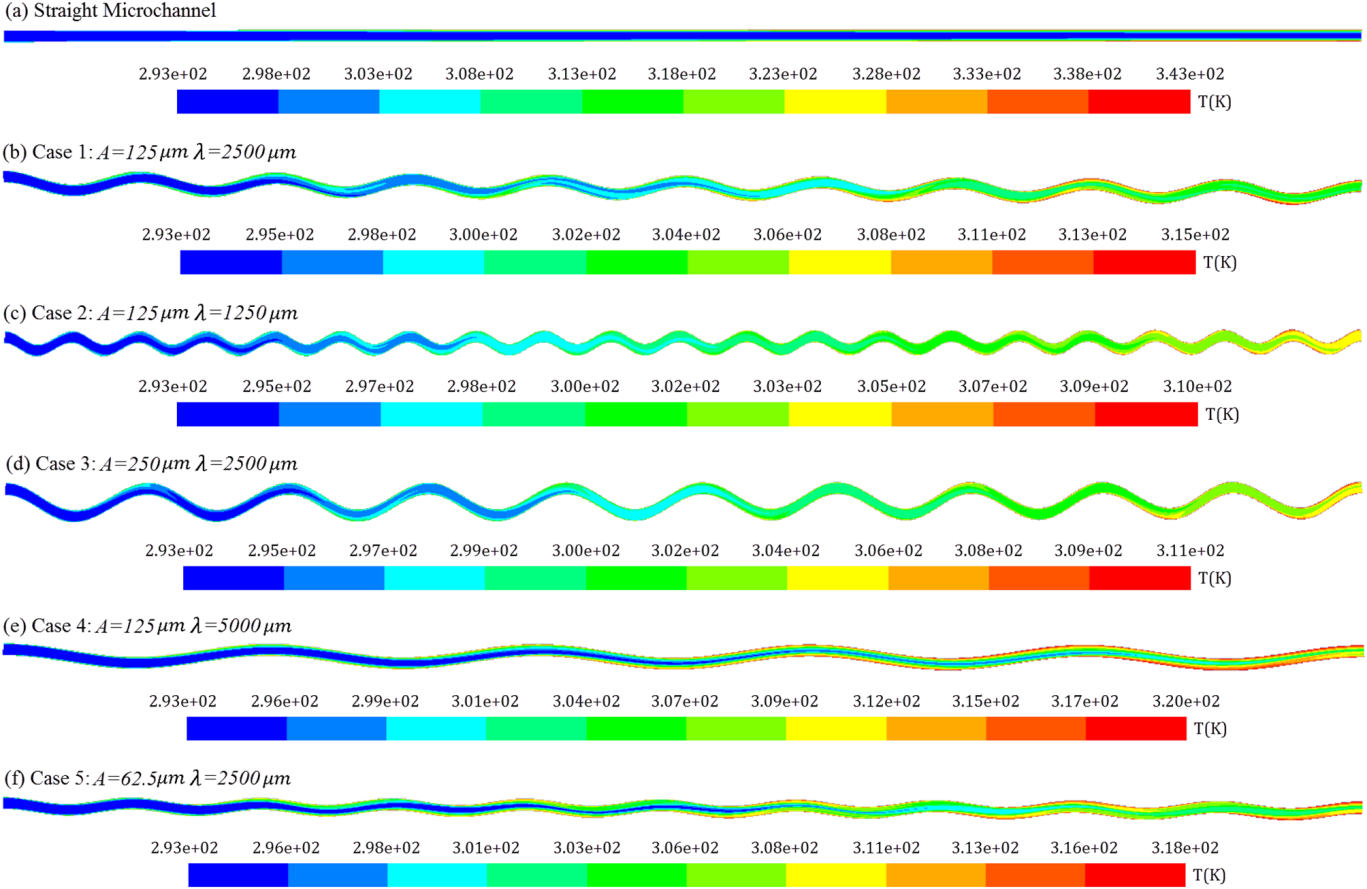
Temperature contours along the microchannel length for: (**a**) Straight channel, (**b**) Case 1 (A = 125 μm, λ = 2500 μm), (**c**) Case 2 (A = 125 μm, λ = 1250 μm), (**d**) Case 3 (A = 250 μm, λ = 2500 μm), (**e**) Case 4 (A = 125 μm, λ = 5000 μm), and (**f**) Case 5 (A = 62.5 μm, λ = 2500 μm).

**Figure 9:**
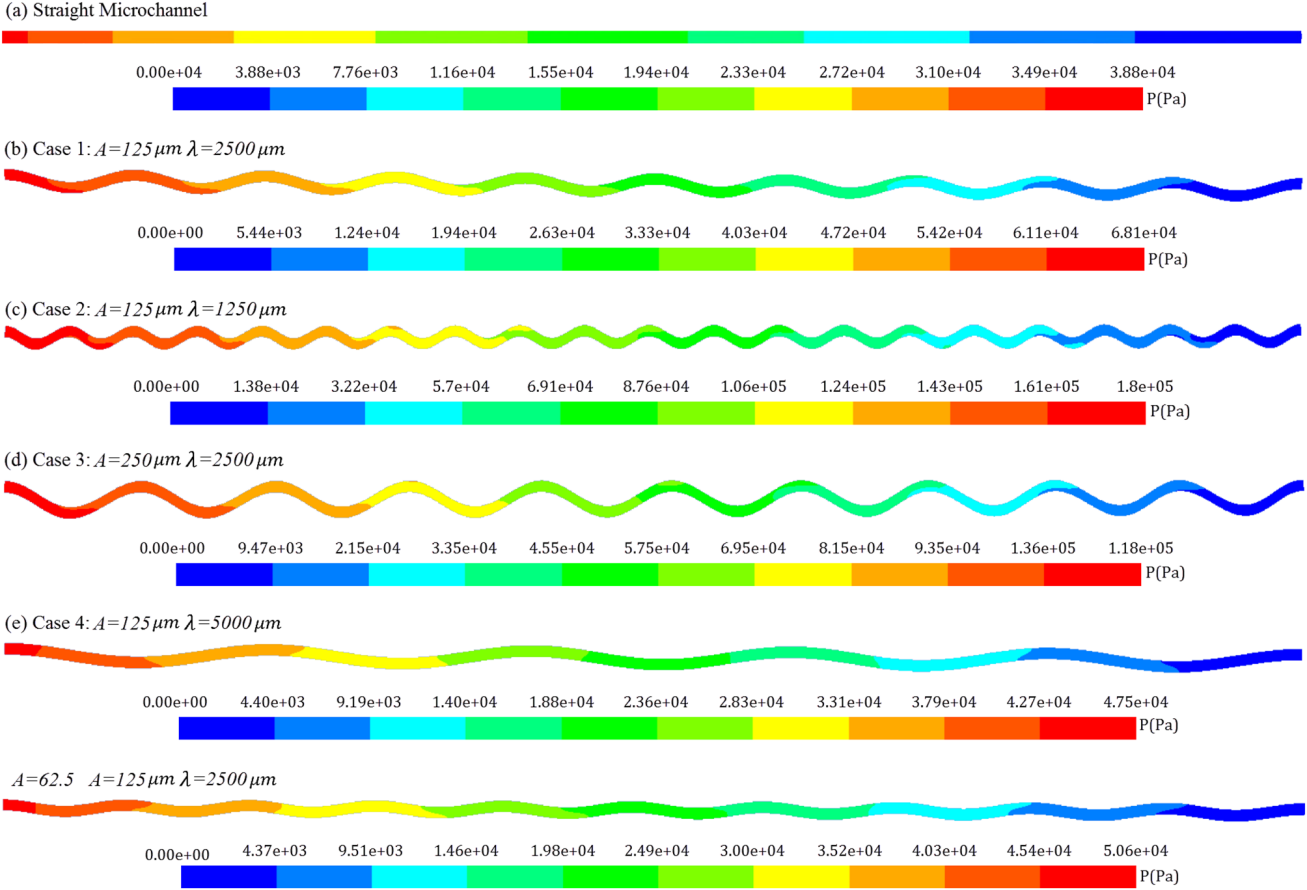
Pressure contours along the microchannel length for: (**a**) Straight channel, (**b**) Case 1 (A = 125 μm, λ = 2500 μm), (**c**) Case 2 (A = 125 μm, λ = 1250 μm), (**d**) Case 3 (A = 250 μm, λ = 2500 μm), (**e**) Case 4 (A = 125 μm, λ = 5000 μm), and (**f**) Case 5 (A = 62.5 μm, λ = 2500 μm).

Temperature and pressure contours along the microchannel length are shown in Figs. 8 and 9 at the Reynolds number of 900. According to the explanations given for the diagrams illustrated in Fig. 7 can be confirmed with these contours. Figure 8 shows that in wavy microchannels, the maximum temperature is lower than the straight microchannel and also the difference between the maximum and minimum temperature is lower in wavy cases. Due to the fluid mixing in wavy microchannels, they have a more uniform temperature field than straight microchannels, and the difference between the temperature of the fluid in the center of the channel and the fluid at the boundary of the channel wall is lower in wavy cases. This issue confirms a higher heat transfer coefficient and Nusselt number and becomes more significant as the amplitude to wavelength ratio increases. Also, according to Fig. 9, it is clear that the pumping power for wavy microchannels is higher than straight microchannels, and with increasing the amplitude and decreasing the wavelength, the pumping power also increases. Therefore, enhancement of the heat transfer in wavy microchannels, also accompanied with more pumping power.

**Figure 10:**
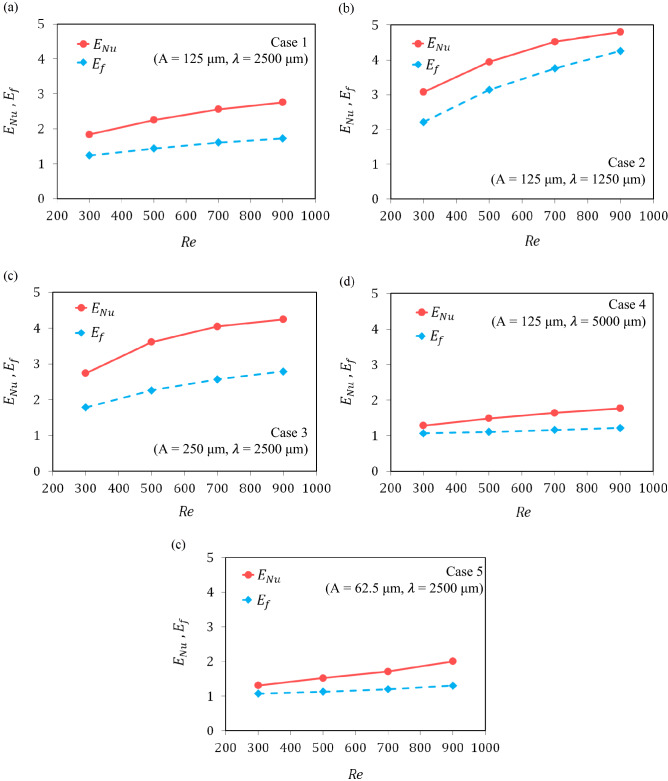
The Nusselt number enhancement and the friction factor enhancement for: (**a**) Case 1 (A = 125 μm, λ = 2500 μm), (**b**) Case 2 (A = 125 μm, λ = 1250 μm), (**c**) Case 3 (A = 250 μm, λ = 2500 μm), (**d**) Case 4 (A = 125 μm, λ = 5000 μm), and (**e**) Case 5 (A = 62.5 μm, λ = 2500 μm).

The two essential parameters in estimating the rate of increase in heat transfer and pressure drop in the microchannels are the parameter of Nusselt number enhancement and the friction factor enhancement, which shows the increases in these parameters in the microchannel respect to the reference microchannel. In the present study, these two parameters are presented for the introduced wavy cases, relative to the straight microchannel. As shown in Fig. 10, in the studied range of the amplitude and wavelength of wavy geometry, for each of the five wavy geometries, the increase in the Nusselt number is higher than the increase in the friction factor, and the highest increase in these two parameters is respectively related to cases 2, 3, 1, 5, and 4. E.g, for Reynolds number of 500, the Nusselt number enhanced by 3.95, 3.62, 2.25, 1.52, and 1.42, times and the friction coefficient increased by 3.15, 2.26, 1.44, 1.13, and 1.11 times for cases 2, 3, 1, 5, and 4, respectively.

Another point is that with the increase of the Reynolds number, the difference between these parameters increase, or in other words, the effect of the heat transfer enhancement is more noticeable than the effect of the pressure drop penalty. Nevertheless, for case 2, this is not true and given the Fig. 10b, the difference between the two parameters is decreasing with the Reynolds number increment from 300 to 900. This could be due to the increase in the number of curved zones and the consequent increase in pressure drop in this geometry due to the decrease in wavelength. This shows that although in the studied range, the heat transfer enhancement is still more significant than the pressure drop penalty. Nonetheless, with further increase in the Reynolds number, it is possible that in some cases, such as case 2, the pressure drop will overcome the heat transfer. This has been reported in other studies that in the wavy microchannels, the heat transfer does not always overcome the pressure drop.

### The relation between the intensity of the transverse flow, heat transfer and the Dean number

**Figure 11:**
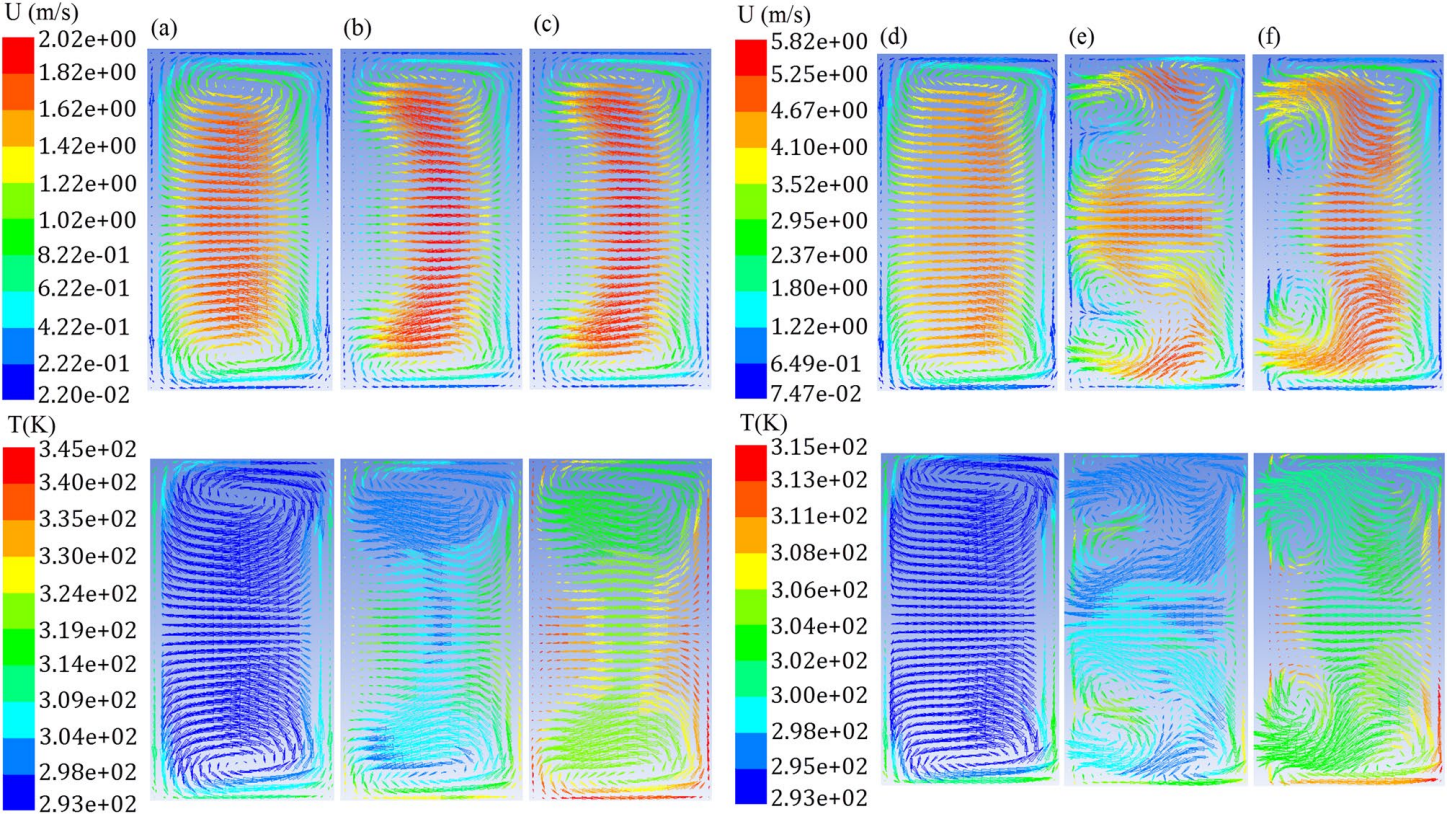
Velocity and temperature distribution with representing velocity vectors for wavy case 1 (A = 125 μm, λ = 2500 μm), (**a**–**c**) at longitudinal distance of 1.25 mm, 11.25 mm,and 23.75 mm from microchannel inlet and Re = 300, and (**d**–**f**) at longitudinal distance of 1.25 mm, 11.25 mm ,and 23.75 mm from microchannel inlet and Re = 900.

**Figure 12:**
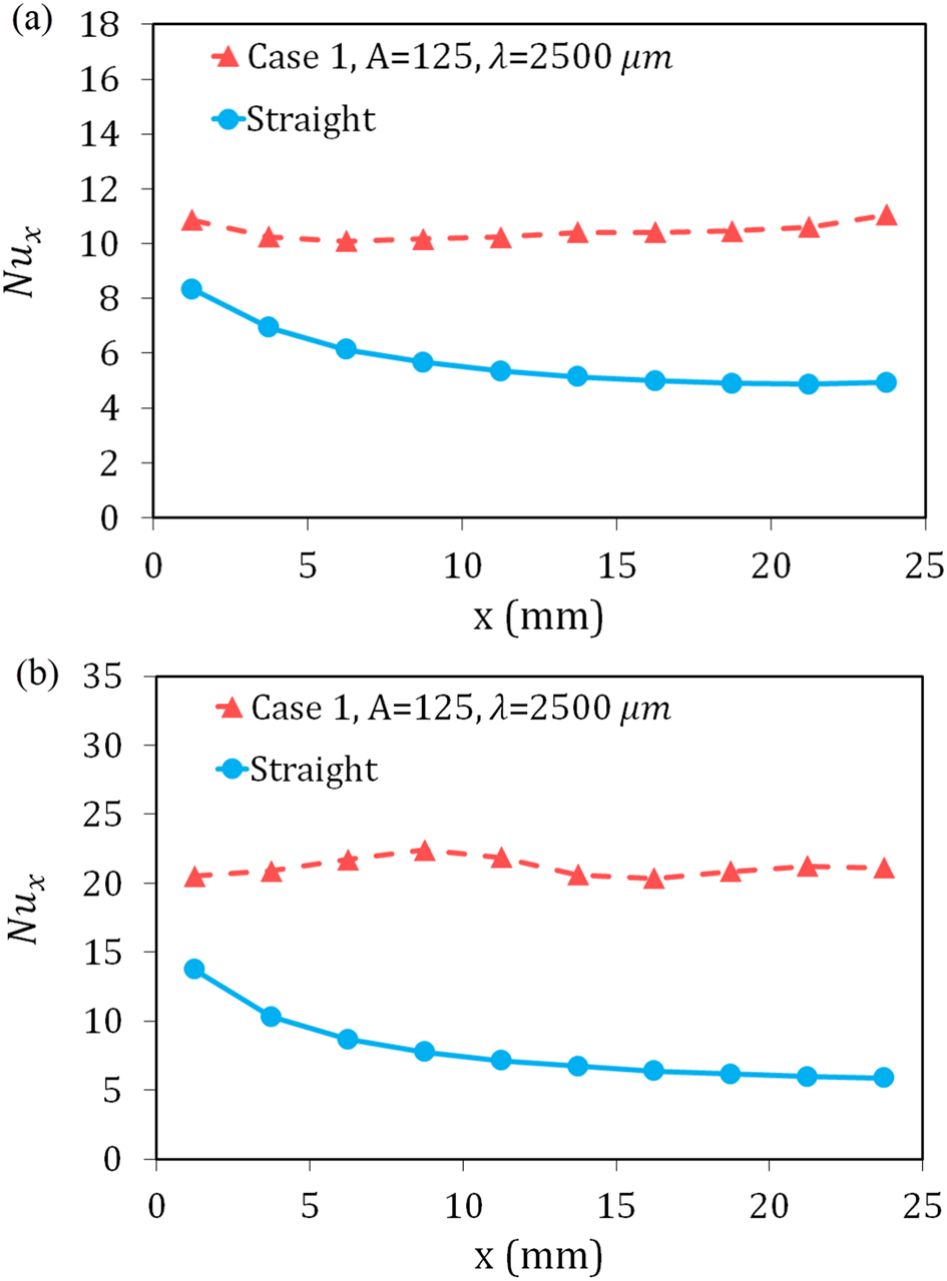
The local Nusselt number for the wavy case 1, and straight microchannel, (**a**) Re = 300, and (**b**) Re = 900.

When a fluid passes through a periodic curved path such as a wavy channel, secondary flows can be generated at the cross-sectional area of ​​the channels due to successive changes in the flow path. This transverse flow, increases fluid mixing and fluid-wall interaction, reducing the temperature difference between fluid and wall, and as a result, heat transfer enhances. By examining the velocity vectors at the cross-sectional area of ​​the wavy microchannels along the channel, it is concluded that the number and shape of the Dean vortices along the flow path are constantly changing due to the oscillating nature of these geometries. For instance, Fig. 11 shows the velocity and temperature distribution at the three cross-sections of the beginning, middle, and end of the microchannel, for the reference wavy geometry at the Reynolds numbers of 300 and 900. As can be inferred, at the Reynolds number of 300, the vortices slightly change along the flow path, whereas as the Reynolds number increases to 900, the number and intensity of the Dean vortices change significantly. This phenomenon permanently disrupts the boundary layer and prevents it from expanding, which justifies higher heat transfer for these geometries. The effect of this phenomenon on augmenting the heat transfer can also be seen by examining the local Nusselt number (Fig. 12).

Figure 12 compares the variations of the local Nusselt number at two Reynolds numbers of 300 and 900 for the reference wavy geometry and straight geometry. Given the figure, it is deduced that the Nusselt number for straight geometry has decreased due to the increase in the thickness of the boundary layers along the microchannel, so that it is decreased by 40% and 57% for Re = 300 and Re = 900, respectively. But for the wavy geometry, the change in the Nusselt number has taken place in a small range and has been almost constant along the microchannel, and there is no noticeable decrease in the Nusselt number for all of the studied cross-sections.

**Figure 13:**
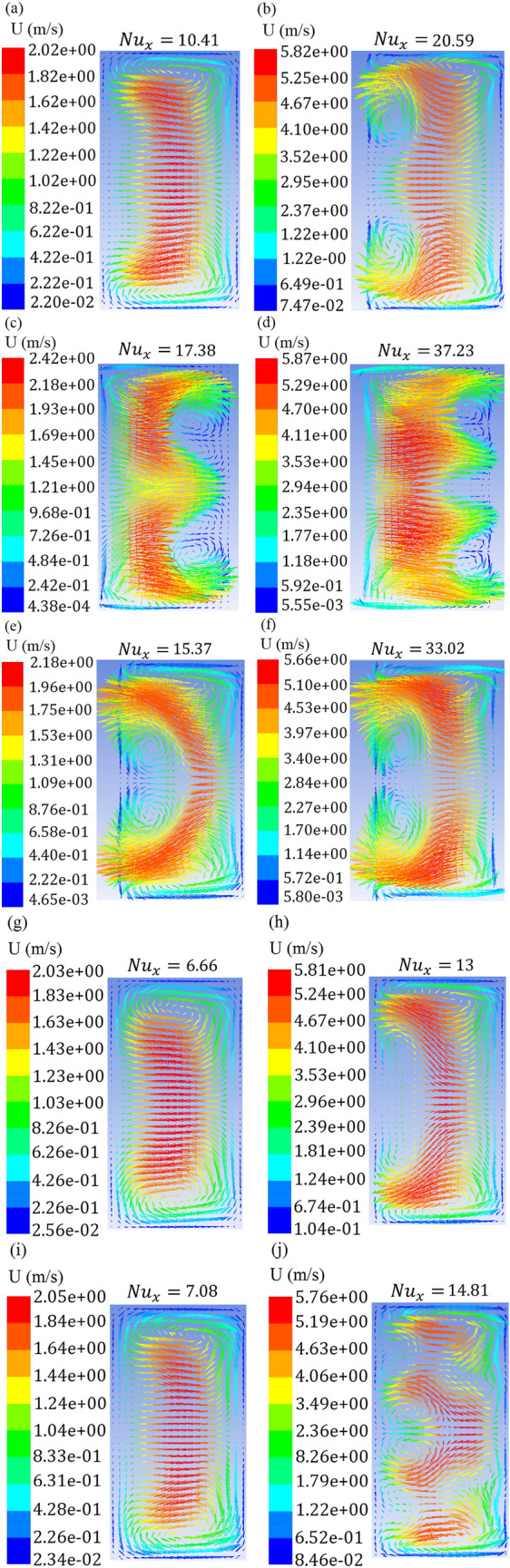
Velocity vectors for (**a**) Case 1, Re = 300, (**b**) Case 1, Re = 900, (**c**) Case 2, Re = 300, (**d**) Case 2, Re = 900, (**e**) Case 3, Re = 300, (**f**) Case 3, Re = 900, (**g**) Case 4, Re = 300, (**h**) Case 4, Re = 900, (**i**) Case 5, Re = 300, (**j**) Case 5, Re = 900.

The effect of the Reynolds number and geometrical parameters on the formation of Dean vortices as well as its relationship with the Dean number is the other interesting points. Figure 13 illustrates the velocity vectors at the cross-sections for the wavy cases at two Reynolds numbers of 300 and 900 (The minimum and maximum of the studied Reynolds number). The local Nusselt number of each case also is given at the top of its velocity vectors. According to Fig. 13, with the Reynolds number increment from 300 to 900, the intensity of the vortices formed at the cross-section of ​​the channels increased for all 5 cases, which led to an increase in the momentum exchange rate of the cold fluid in the center of the channel and hot fluid near the wall and thereby enhanced heat transfer. As can be seen in the definition of the Dean number, it is directly related to the Reynolds number, and increasing the intensity of the Dean vortices as the Reynolds number get higher is justified by the definition of the Dean number.

It can be seen in Fig. 13 for the higher amplitude to wavelength ratio in case 2 and case 3, the intensity of vortices has increased compared to case 1, and also in case 4 and case 5 with decreasing the amplitude to wavelength ratio, the intensity of vortices has decreased compared to case 1. According to the Nusselt number diagram and the friction factor presented in the previous section and velocity vector analyses, it is concluded that there is a strong relationship between physics of fluid flow in these geometries and thermal and hydraulic performance. Nevertheless, why does increasing the amplitude and decreasing the wavelength, or in other words, increasing the amplitude to wavelength ratio in wavy geometries, increase the intensity of the vortices and increase the heat transfer. In fact, why change the geometric parameters of amplitude and wavelength significantly affects the physics of the flow. By examining the definition of the Dean number and considering the constant hydraulic diameter of the channel in all of the studied geometries, it is inferred that the only effective geometric parameter is the radius of curvature of the flow path. The radius of curvature of all five wavy geometries is calculated at their extremum and is demonstrated in Table 2. As in Table 2 has been shown, case 2 and case 3 have a smaller radius of curvature than the reference wavy geometry, and as a result, have a larger Dean number, and therefore the intensity of the vortices in these geometries has increased. For case 4 and case 5, it is also clear that the radius of curvature is greater, and therefore the Dean number is smaller than the reference wavy geometry. Therefore, as the amplitude to wavelength ratio increases, the radius of curvature decreases and thus the Dean number and heat transfer increases.

**Figure 14:**
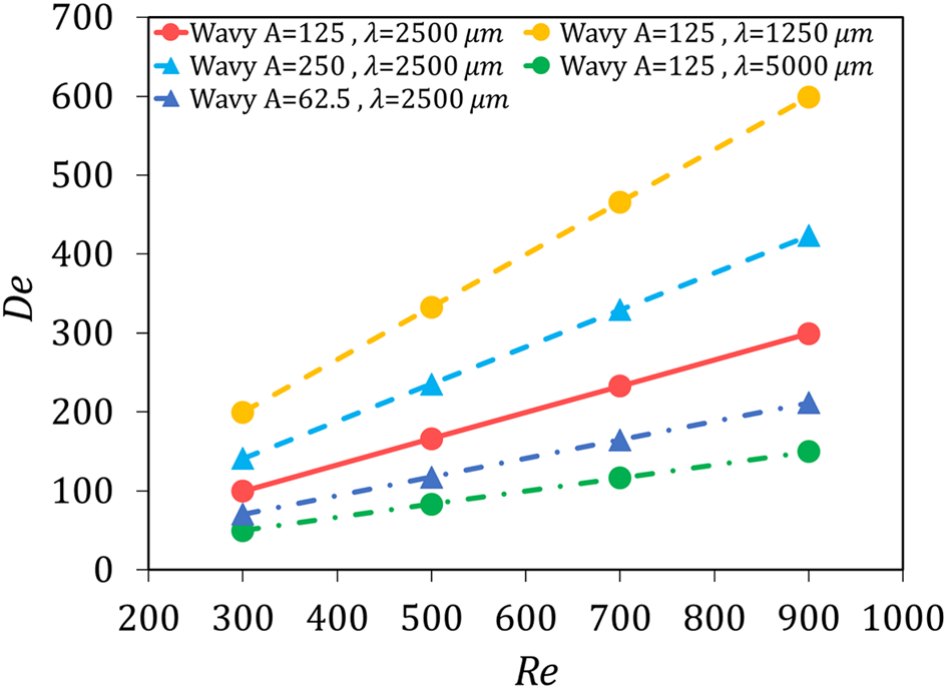
Dean number versus Reynolds number.

Figure 14 shows a diagram of the Dean number according to the Reynolds number. As depicted in Fig. 14, case 2 has the highest values of the Dean number, followed by cases 3, 1, 5, and 4, respectively. In an equal amplitude to wavelength ratio (case 2 with case 3 and case 4 with case 5), the geometry that has a smaller amplitude and wavelength, has a smaller radius of curvature and therefore a larger Dean number. Therefore, wavy cases 2 and 5 have better thermal performance than cases 3 and 4, respectively. Considering the definition of the Dean number, in a constant hydraulic diameter and radius of curvature, it changes linearly with the Reynolds number, and the slope of the diagram for geometry with lower radius of curvature is greater, which justifies the augmentation of the secondary flow, and the higher slope of the Nusselt number versus Reynolds number diagram for these geometries.

It is practical to compare the velocity components at the cross-section of the wavy cases to establish the relationship between the Dean number, transverse velocities, and the ratio of transverse velocities to axial velocity and its role in the formation of the secondary flow and amplification of the heat transfer. Tables 5 and 6 show the geometrical specifications, Dean number, velocity components, and the ratio of transverse to the axial velocity of the wavy cases at two Reynolds numbers of 300, and 900 in the middle of the channel length. As in Table 5 can be seen, the mean values of the velocity components, and in Table 6, the maximum values of the velocity components are considered. The reason for the simultaneous study of average and maximum velocities is to examine whether there is a difference in velocity analysis in these two modes. The second reason is that some changes such as changes in velocity component in “z” direction, which is not sensible in the average mode, can be traced in the maximum mode.

**Table 5:** Velocity components and ratios for wavy cases in average velocity mode.

Case no.	*A* (μm)	$\lambda$ (μm)	$R_{C}$ (μm)	Dean number	$u_{avg}$ (m/s)	$v_{avg}$ (m/s)	$w_{avg}$ (m/s)	$v_{avg} /u_{avg}$	$w_{avg} /u_{avg}$
**Re = 300**									
Case 1	125	2500	1266.5	99.84	1.073	0.047	7.56E−08	0.044	7.08E−08
Case 2	125	1250	316.6	199.67	1.073	0.149	2.11E−07	0.139	1.97E−07
Case 3	250	2500	633.2	141.18	1.073	0.091	5.63E−07	0.085	5.24E−07
Case 4	125	5000	5066.1	49.91	1.073	0.016	1.04E−09	0.015	9.71E−10
Case 5	62.5	2500	2533.1	70.59	1.073	0.028	1.63E−06	0.026	1.52E−06
**Re = 900**									
Case 1	125	2500	1266.5	299.5	3.219	0.137	2.04E−06	0.043	6.37E−07
Case 2	125	1250	316.6	599.01	3.219	0.420	1.64E−06	0.130	5.09E−07
Case 3	250	2500	633.2	423.56	3.219	0.246	8.50E−06	0.076	2.64E−06
Case 4	125	5000	5066.1	149.75	3.219	0.046	5.24E−07	0.014	1.63E−07
Case 5	62.5	2500	2533.1	211.78	3.219	0.082	2.17E−06	0.025	6.74E−07

**Table 6:** Velocity components and velocity ratios for wavy cases in maximum velocity mode.

Case no.	*A* (μm)	$\lambda$(μm)	$R_{C}$(μm)	Dean number	$u_{\max }$(m/s)	$v_{\max }$(m/s)	$w_{\max }$(m/s)	$v_{\max } /u_{\max }$	$w_{\max } /u_{\max }$
**Re = 300**									
Case 1	125	2500	1266.5	99.84	1.96	0.135	0.101	0.069	0.052
Case 2	125	1250	316.6	199.67	2.25	0.450	0.195	0.2	0.087
Case 3	250	2500	633.2	141.18	1.96	0.244	0.221	0.125	0.113
Case 4	125	5000	5066.1	49.91	2	0.064	0.050	0.032	0.025
Case 5	62.5	2500	2533.1	70.59	2.04	0.076	0.064	0.037	0.031
**Re = 900**									
Case 1	125	2500	1266.5	299.5	5.25	0.532	0.408	0.101	0.078
Case 2	125	1250	316.6	599.01	5.60	0.953	0.444	0.170	0.079
Case 3	250	2500	633.2	423.56	5.14	0.838	0.755	0.163	0.147
Case 4	125	5000	5066.1	149.75	5.60	0.235	0.144	0.042	0.026
Case 5	62.5	2500	2533.1	211.78	5.71	0.256	0.181	0.045	0.032

According to the mentioned tables, in both the average velocity and maximum velocity modes, the velocity values ​​along the width of the channel cross-section (*v*) are higher for geometries that have a smaller radius of curvature and have a higher Dean number, but this is not always true for velocity along the channel height. For instance, case 2 has a higher Dean number than case 3, but its “z” velocity component (*w*) is smaller than that of case 3. This is because most of the velocity vectors in the secondary flow are in the direction of the channel width, and according to the Figs. 11 and 13, the “z” components (*w*) is created as a consequence of the collision of the “y” components (*v*) with the channel wall and then with the formation of vortices. Therefore, the “y” components play a key role in the formation of the transverse flow. When the “y” component is traced, it is inferred that it has conformity with the increase of the Dean number. Geometries that have a smaller radius of curvature and a higher Dean number, have a higher “y” component, and also have a higher ratio of “y” to “x” component (*v*/*u*). This means that a larger portion of the flow is converted to the secondary flow, that improves the heat transfer between the fluid and the wall. As a result, similar to what was observed in the analysis of the Nusselt number, case 2 has the highest values of “y” component followed by case 3, case 1, case 5, and case 4, respectively.

Another point is that in the average velocity mode, as the velocity vectors are taken on average, and since the secondary flow at the cross-section of the channels is usually symmetrically formed, the order of the average velocity in the “z” direction becomes very small. However, in the maximum mode, since only the maximum vector is reported, this issue is not seen.

### Performance evaluation criterion

**Figure 15:**
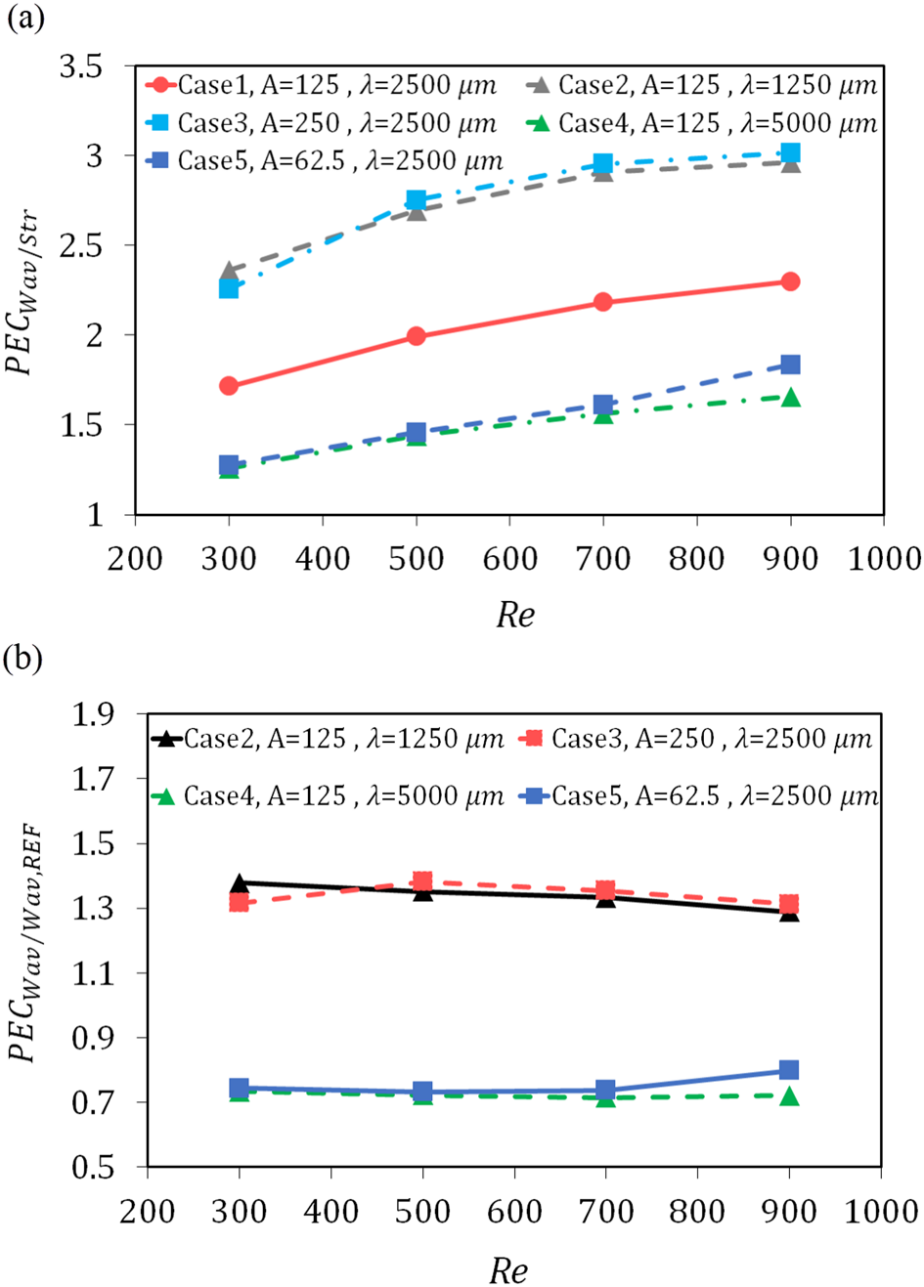
Performance evaluation criterion versus Reynolds number: (**a**) When straight pattern considered as the reference, and (**b**) when wavy case 1 considered as the reference geometry.

The performance evaluation criterion (PEC) is one of the critical parameters to assess the thermal–hydraulic performance of the microchannel heat sink. As depicted in Fig. 15a, when straight geometry is considered as the reference geometry, the PEC values ​​for all five wavy geometries are greater than 1, indicating that all of the studied geometries have a better PEC than that of straight geometry. Also, as the Reynolds number increases, the performance evaluation criterion increases, and the slope of its increase decreases. Wavy cases with the same amplitude to wavelength ratio have PEC values ​​close to each other and have remarkable difference with the reference wavy geometry. This shows that the parameter amplitude to wavelength ratio plays an essential role in the thermal–hydraulic performance of these geometries. Wavy case 3 has the highest PEC values, followed by cases 2, 1, 5, and 4, respectively.

When the wavy case 1 is considered as the reference for comparing thermal–hydraulic performance, according to Fig. 15b, it is clear that PEC values ​​do not always increase for the higher Reynolds number, and the maximum value of PEC is 1.38 at the Reynolds number of 500 and for the wavy case 3. The slope of the diagram for the wavy case 2 is negative, and for the wavy case 3 to Re = 500 is positive and then negative. Therefore, it is concluded that in the range of higher Reynolds numbers, as the amplitude to wavelength ratio increases, the pressure drop in these geometries may overcome the heat transfer and thus show a weaker overall performance than the reference wavy geometry. However, in the present study, the wavy case 3 with the amplitude of 250 μm and wavelength of 2500 μm had the best PEC.

### Comparison of the optimal wavy channel with the straight channel in variable flux conditions

Electronic chips may be exposed to different heat fluxes that result from user activities. The heat flux fluctuations cause to create hotspots in the chip, which leads to damage to the device. Therefore, choosing the most appropriate cooling system plays a significant role in prohibiting system damages. One of the most critical features of an efficient microchannel heat sink is removing different heat fluxes instantly and preventing unallowable temperature. With introducing the best wavy microchannel in “Performance evaluation criterion”, a comparison is conducted between the optimal case (case 3) and straight one in the Reynolds number of 900. Heat fluxes of 80, 120, 160, 200, and 240 W/cm^2^ are applied. To analyze microchannels reactions, the applied heat fluxes are increased by time.

First, all mentioned heat fluxes were applied to the straight and wavy case 3 at the steady-state condition to indicate the maximum created temperature in the microchannel. All maximum temperatures occurred at the bottom surface of the microchannel that is reported in Table 7. At the heat flux of 120 W/cm^2^, the maximum temperature of straight microchannel reaches 367.25 K, which is close to the boiling point of water. Therefore, straight microchannel of the present study could not be utilized at higher heat flux at Reynolds number of 900. Whereas the maximum solid temperature in case 3 even at the heat flux of 240 W/cm^2^, is 346.30 K, which shows that at an equal Reynolds number, the maximum heat flux that could remove by wavy microchannel is much higher than that of straight microchannel. Besides, by increasing the heat flux from 80 W/cm^2^ to 120 W/cm^2^, temperature increment of straight microchannel is 24.7 K, while for wavy microchannel is 8.86 K, which is much lower. As a result, it can be inferred that at high heat fluxes, wavy microchannel perform much better than straight microchannel and could prevent creating hotspots in the chip.

**Table 7:** The maximum fluid and solid temperatures at the different heat fluxes for cases 3 and Straight microchannels.

Heat flux (W/cm^2^)	Max. temp of solid in straight (K)	Max. temp of solid in case 3 (K)
80	342.55	310.87
120	367.25	319.73
160	***	328.57
200	***	337.44
240	***	346.30

**Figure 16:**
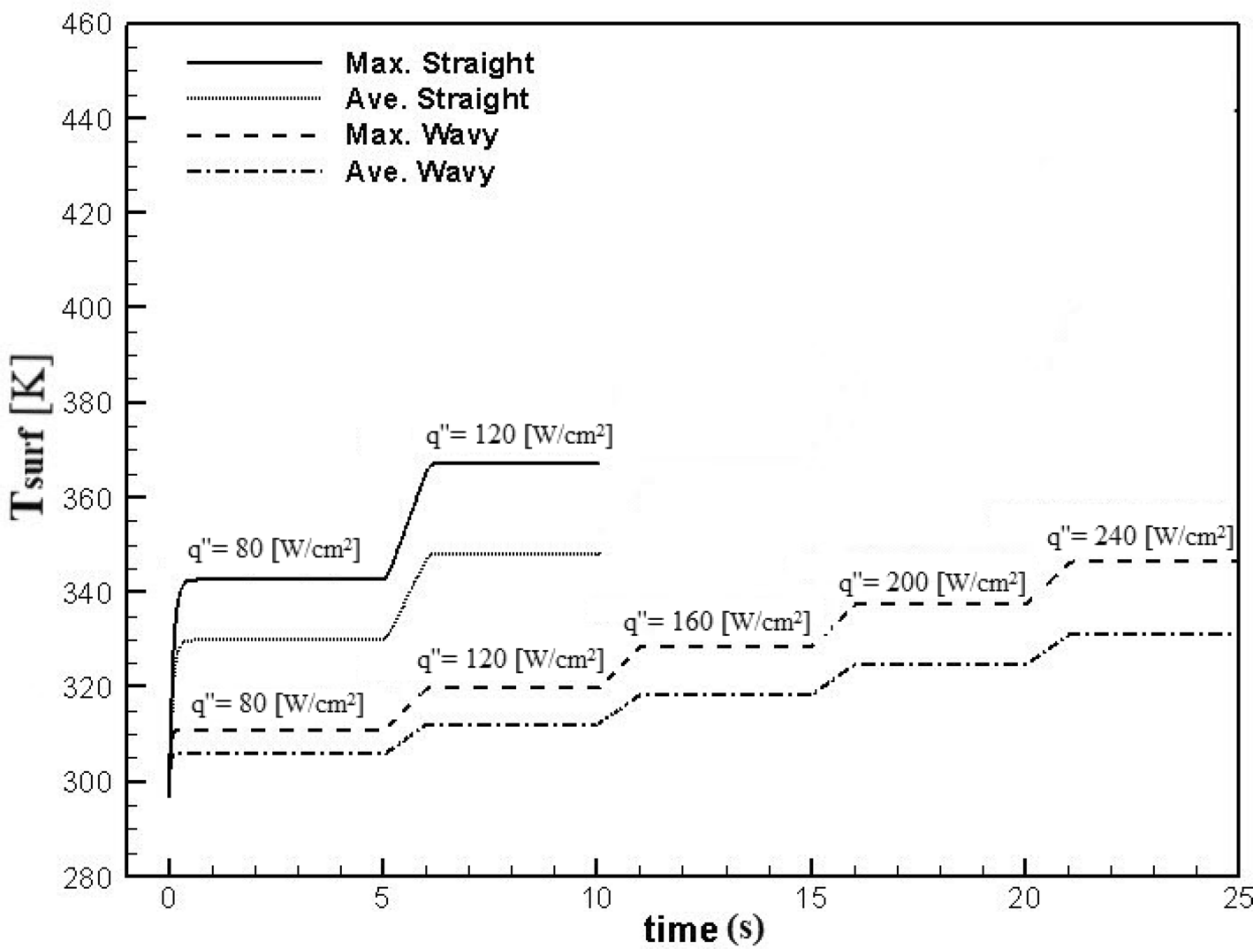
Maximum and average surface temperature versus time for the wavy case 3 and straight microchannel at Re = 900 for different heat fluxes.

After simulating microchannels in steady-state conditions for all mentioned heat fluxes, the simulation was conducted in the transient state to estimate the cooling time by microchannels. The procedure is that at the first moment, a heat flux of 80 W/cm^2^ is applied to the microchannels. When the surface temperature of the microchannel is constant and after five seconds, a heat flux of 120 W/cm^2^ is suddenly applied and continues in the same way until a heat flux of 240 W/cm^2^ is applied at the bottom surface of the wavy microchannel. As mentioned before, this procedure is applied for straight microchannel until heat flux of 120 W/cm^2^. The results of this comparison are illustrated in Fig. 16 for maximum and average surface temperatures. The first point that can be seen in Fig. 16 is the significantly higher maximum temperature in the straight channel compared to the wavy channel, which again shows the better performance of the wavy microchannel. The second point is the meaningful difference between the maximum surface temperature and its average in the straight microchannel, much less visible in the wavy case. When the average temperature of electronic chips is reported at any moment, there may be hotspots on the surface of the chip that are hidden in the average temperature report and cause damage to the chip. The minor difference between the maximum and average surface temperatures means that the cooling system creates a more uniform temperature distribution in the chip and prevents hotspots. Therefore, according to Fig. 16, it is clear that the wavy microchannel has responded well to the increase in temperature and has created a much more uniform surface temperature. This ensures no significant temperature difference between the different parts of the electronic chip, and consequently prevents damage.

According to the results, the microchannel takes 1.5 to 2 s to damps the temperature rise caused by a sudden increase in heat flux. In cases where this temperature rise enters the unauthorized range, to ensure that the chip is not damaged, by placing a sensor on the electronic system, it can order the entire system to shut down as soon as the temperature rises. As a second way, it can order the pump to increase the flow rate rapidly, which could prevent the temperature increment.

## Conclusions

The thermal–hydraulic performance of five wavy geometries is investigated numerically over various amplitudes and wavelengths in the laminar flow range. The significant results of this paper are stated as follows:

1. The average Nusselt number and the friction factor were examined, and all of the wavy geometries had higher heat transfer and pressure drop than straight geometry. Wavy case 2 with the amplitude of 125 μm and the wavelength of 1250 μm had the highest values ​​of these two parameters, followed by cases 3, 1, 5, and 4, respectively. The increase in the Nusselt number of these five geometries was higher than the increase in the pressure drops, so that, for instance, at the Reynolds number of 500, the Nusselt number enhanced by 3.95, 3.62, 2.25, 1.52 and 1.42 times and the friction coefficient increased by 3.15, 2.26, 1.44, 1.13, and 1.11 times for cases 2, 3, 1, 5, and 4, respectively. It was also found that in the studied range of amplitude, wavelength, and flow rate, the effect of wavelength change was greater than amplitude change on increasing or decreasing heat transfer and pressure drop in these geometries. Based on the obtained results, if the pressure drop in the wavy geometry is ignored, the wavy case 2 with the amplitude of 125 μm and the wavelength of 1250 μm is suggested to achieving the highest heat transfer.
2. By evaluating the velocity vectors at the cross-sectional area of the wavy geometries, it was highlighted that the shape and number of Dean vortices change due to the oscillating nature of these geometries along the flow path. This constant change causes the boundary layer to not reach its developed state and thus increases heat transfer. The effect of this phenomenon is evident in the local Nusselt number of these geometries so that it fluctuates along the flow path in a small range and remains almost constant.
3. With the increase in the Reynolds number and the amplitude to wavelength ratio, it was observed that the intensity of the Dean vortices increased. According to the definition of the Dean number, it is clear that in the same hydraulic diameter, the two parameters of the Reynolds number and the radius of curvature affect the Dean number and the intensity of the vortices. Due to the direct relation of the Dean number with the Reynolds number and the inverse relation with the radius of curvature, increment of the Reynolds number and decrement of the radius of curvature lead to an increase of the Dean number and thus increase the intensity of the vortices and heat transfer. In fact, it was found that wavy geometries that have a larger amplitude to wavelength ratio have smaller radius of curvature, so that two cases 2, and 3, showed the highest values ​​of Dean number and highest heat transfer. In a certain amplitude to wavelength ratio, the geometry that has a smaller amplitude and wavelength, has a larger Dean number and higher heat transfer. Therefore, in the applicable samples, to achieving lower radius of curvature, and thereby, higher Dean number and heat transfer coefficient, it is recommended to choose lower amplitude and wavelength and larger amplitude to wavelength ratio as far as possible and according to the fabrication constraints.
4. With velocity components analysis, it was found that the geometries that have a smaller radius of curvature and a higher Dean number, have a higher “y” velocity component, and also have a higher ratio of “y” to “x” component (*v*/*u*). This higher ratio means that a larger portion of the flow is converted to the secondary flow, and thereby enhances the heat transfer in them.
5. Assessment of the performance evaluation criterion (PEC) showed that all of the studied wavy geometries have better thermal–hydraulic performance than straight geometry. Geometries with the same amplitude to wavelength ratio have PEC values close to each other, indicating that this geometrical parameter plays a critical role in the performance of these geometries. Finally, wavy case 3 with the amplitude of 250 μm and the wavelength of 2500 μm is introduced as the best geometry from the thermal–hydraulic performance point of view.
6. Investigation of temporal variable flux condition for the best wavy and straight cases illustrated that the wavy microchannel creates much more uniform temperature distribution and prevents hotspots generation. Also, by time analysis, it was found that it takes 1.5 to 2 s for microchannels to damp the temperature increment of the electronic chip, thus in cases where this temperature rise enters the unallowable range, to ensure that the chip does not damage, by considering a sensor on the electronic system, it can order the entire system to shut down as soon as the temperature rises, or increases the flow rate to prevent the temperature increment and chip damage.

The results of the current study, in an applicable range of the Reynolds numbers (300 to 900), amplitude (62.5 to 250 μm), and wavelength (1250 to 5000 μm), are a valuable reference for achieving a high heat transfer rate to cooling the high heat flux electronic chips. It is possible to increase the heat transfer to the desired level by selecting the appropriate amplitude to wavelength ratio and radius of curvature in each study range so that allowing the chip to run in higher heat fluxes in a way that pumping power also overcome the pressure drop by using the results of this paper.

## Abbreviations

$A_{b}$: Base area of channel (m^2^)
$A_{i}$: Fluid–solid interface area (m^2^)
$A_{c}$: Cross-section area of channel (m^2^)
$C_{{P_{f} }}$: Specific heat of fluid (J/kg K)
$C_{{P_{s} }}$: Specific heat of solid (J/kg K)
$$De$$: Dean number
$$f$$: Friction factor
$$H_{ch}$$: Channel height (m)
$h_{x}$: Local heat transfer coefficient (W/m^2^ K)
$k_{s}$: Thermal conductivity of solid (W/m K)
$k_{f}$: Thermal conductivity of fluid (W/m K)
$$L_{ch}$$: Channel length (m)
$n$: Perpendicular direction to the surface
$Nu_{avg}$: Average Nusselt number
$Nu_{x}$: Local Nusselt number
$P_{c}$: Perimeter of microchannel cross section (m)
$p_{0}$: Atmospheric pressure (Pa)
$\Delta P$: Pressure drop (Pa)
$$q^{\prime\prime}$$: Heat flux (W/m^2^)
$R_{C}$: Radius of curvature (m)
$${\text{Re}}$$: Reynolds number
$$T_{f}$$: Fluid temperature (K)
$$T_{s}$$: Solid temperature (K)
$$T_{Surf}$$: Surface temperature (K)
$T_{f,in}$: Inlet fluid temperature (K)
$T_{W,x}$: Local channel wall temperature (K)
$T_{f,x}$: Local fluid bulk temperature (K)
$$\vec{U}$$: Velocity vector (m/s)
$u$: Velocity component in x direction (m/s)
$u_{in}$: Inlet fluid velocity (m/s)
$u_{avg}$: Average fluid longitudinal velocity (m/s)
$v$: Velocity component in y direction (m/s)
$w$: Velocity component in z direction (m/s)
$$W_{ch}$$: Channel width (m)
$$W_{s}$$: Substrate width (m)
$$W_{t}$$: Substrate thickness (m)

## Greek symbols

$\rho_{f}$: Fluid density (kg/m^3^)
$\rho_{s}$: Solid density (kg/m^3^)
$\mu_{f}$: Fluid dynamic viscosity (kg/m s)
$\lambda$: Wavelength of the wavy geometries (m)

## Subscripts

$$avg$$: Average
$$ch$$: Channel
$$f$$: Fluid
$$in$$: Inlet
$$\max$$: Maximum
$$s$$: Solid
$$W$$: Wall
